# Recent advances in layered double hydroxides applied to photoprotection

**DOI:** 10.31744/einstein_journal/2019RW4456

**Published:** 2019-02-14

**Authors:** Tamires Andrade da Silva, Tamares Andrade da Silva, Ticiano Gomes do Nascimento, Rebeca Evahides Yatsuzuka, Luciano Aparecido Meireles Grillo, Camila Braga Dornelas

**Affiliations:** 1 Universidade Federal de Alagoas, Maceió, AL, Brazil.; 2 Universidade de São Paulo, São Paulo, SP, Brazil.

**Keywords:** Nanotechnology, Nanostructures, Clay, Solar radiation, Ultraviolet filters, Nanotecnologia, Nanoestruturas, Argila, Radiação solar, Filtros ultravioletas

## Abstract

Layered double hydroxides have received more attention from researchers due to their range of applications, ease of synthesis and low cost of production. With broader knowledge about solar radiation effects on the body, the use of sunscreens has become even more important. The ability of some nanostructures, such as layered double hydroxides, to act as matrices has made it possible to obtain improvements in photoprotective formulations, with solutions to problems caused by radiation and sunscreens. This review article brings together the most recent advances of these clays, the layered double hydroxides, applied to photoprotection.

## RESUMO

Os hidróxidos duplos lamelares vêm ganhando maior atenção de pesquisadores, em decorrência da gama de aplicações, da facilidade de síntese e do baixo custo de produção. Com o crescente conhecimento sobre diversos efeitos da radiação solar sobre o organismo, a utilização de fotoprotetores tem se tornado indispensável. A capacidade de nanoestruturas, como os hidróxidos duplos lamelares, atuarem como matrizes possibilitou melhorias nas formulações fotoprotetoras, tendo em vista os problemas provocados pela radiação, bem como por alguns filtros solares. Este artigo de revisão reúne os avanços mais recentes destas argilas, os hidróxidos duplos lamelares, aplicados à fotoproteção.

## INTRODUCTION

Sunscreens are used to protect skin against ultraviolet (UV) solar radiation, which is harmful to humans. The effects of this radiation range from mild burns to development of skin cancer.^(^ ^1^ ^)^ Some molecules responsible for the absorption of UV rays present in sunscreens have disadvantages, and may even cause damage when used in high concentrations,^(^ ^2^ ^)^ which motivates the investigation of new approaches.

Nanotechnology entails the development, characterization and application of nanometer-scale systems. The targeting and control of the release of active substances on the skin enabled by nanotechnology has become object of studies throughout the world.^(^ ^3^ ^)^ In this context, several types of nanostructures, including solid lipid nanoparticles,^(^ ^4^ ^)^ titanium-dioxide and zinc-oxide nanoparticles,^(^ ^5^ ^)^ polymeric nanoparticles^(^ ^6^ ^)^ and liposomes,^(^ ^7^ ^)^ among others, promise to have substantial scientific and technological impact, primarily due to their particular properties.

The advantages of these systems include chemical and physical improvement of the molecules, contributing to greater stability;^(^ ^8^ ^)^ increased penetration in areas that are difficult to access; the possibility of providing controlled release, avoiding systemic absorption;^(^ ^9^ ^)^ and, finally, unwanted organoleptic properties can be masked.^(^ ^10^ ^)^

In particular, the use of inorganic nanoparticles, especially layered double hydroxides (LDHs), attracts attention from investigators because they present promising properties, including ease of laboratory synthesis, biocompatibility, low toxicity, capacity for significant ion exchange, increased stability of the inserted species, vectorization of active compounds, and possibility of controlled release.^(^ ^11^ ^,^ ^12^ ^)^

## OBJECTIVE

To present concise but detailed information regarding the latest advances in layered double hydroxides in formulations for photoprotection of the face and body skin.

## METHODS

This is a systematic review that addressed the use of LDHs in photoprotective formulations. The following descriptors were searched in the electronic databases ScienceDirect and PubMed: initially, “lamellar clays”, then, “layered double hydroxides”; “layered double hydroxides and sunscreens”, “layered double hydroxides and photoprotection”.

The search for published articles was carried out in February 2018, and covered 2000 to 2018. Publications that did not address the topic investigated and that did not offer concise information regarding the applied methodology and/or results were excluded. Emphasis was placed on original articles. After reading the abstracts, we selected articles for the study, in order to present the most relevant results verified by the authors.

## RESULTS

### Lamellar clays

Clay refers to a natural material, composed of fine-grained minerals that, when moistened with water, shows properties of plasticity.^(^ ^13^ ^)^ Lamellar clays are arranged in crystalline lamellae that represent their conformational structure,^(^ ^14^ ^)^ and have two dimensions on the order of nanometers.^(^ ^15^ ^)^

Among natural anionic clays, natural hydrotalcite, similar in structure to brucite or magnesium hydroxide − Mg(OH)_2_ −, is well known, where the magnesium atoms have an octahedral coordination by means of hydroxyl groups.^(^ ^16^ ^)^ In this context, the LDHs are included.

### Layered double hydroxides

Layered double hydroxides, also called anionic clays, constitute a large family of lamellar solids.^(^ ^16^ ^)^ These compounds are represented by the general formula [M^2+^_1−x_M^3+^_x_(OH)_2_]^−^ (A^n−^)_x/n_·mH_2_O], in which M^2+^ and M^3+^ are divalent and trivalent cations, respectively, which are in the octahedral position in the lamellae of the LDH.^(^ ^17^ ^)^ Divalent cations are isomorphically replaced by trivalent cations, and the lamellae have a positive residual charge. Anions are required to compensate for this charge. Together with water, they promote the stacking of the layers. There is virtually no limitation to the nature of the anions that can compensate for the positive charge on LDH lamellae.^(^ ^18^ ^,^ ^19^ ^)^

These materials can be synthesized in the laboratory by simple methods,^(^ ^10^ ^)^ but some factors must be evaluated, including degree of agitation, speed of addition of one solution to another, final pH of the resulting suspension (for pH-variable methods), temperature of the final solution (usually ambient temperature), and in some cases atmosphere control.^(^ ^19^ ^)^

Ease of manipulation of its properties, the wide range of chemical compositions and the low cost^(^ ^20^ ^,^ ^21^ ^)^ are characteristics that grant LDHs great versatility in diverse fields of application.^(^ ^22^ ^,^ ^23^ ^)^ One application that has been highlighted for the use of LDHs is photoprotection.^(^ ^24^ ^,^ ^25^ ^)^

### Ultraviolet filters intercalated in layered double hydroxides

The incorporation of organic molecules (sunscreens) into two-dimensional inorganic materials LDHs provides an intriguing way to preserve organic molecules, since LDHs prevent direct contact of organic molecules with human skin. Moreover, the anions between the lamellae are connected through electrostatic interaction. Therefore, both chemical stabilization and protection can be acquired.^(^ ^1^ ^,^ ^26^ ^,^ ^27^ ^)^

From the total of articles researched, table 1 shows those that are directly related to the object of study and that are discussed next.

**Table 1:** Articles description

Authors	LDH	UV absorber	Method
Khan et al.^(^ ^1^ ^)^	ZnAl	3,4-dihydroxycinnamic acid (cinnamic acid)	Co-precipitation
		4-hydroxy-3,5-dimethoxycinnamic acid (SA)	
		3-amino-5-trifluoromethylbenzoic acid (FBA)	
Li et al.^(^ ^2^ ^)^	ZnAl	2,4-dihydroxybenzophenone-5-sulfonate	Co-precipitation
Li et al.^(^ ^24^ ^)^	ZnTi	Cinnamic acid	Ion-exchange
Wang et al.^(^ ^25^ ^)^	MgAl	Zinc	Co-precipitation
	ZnAl	Titanium salts	
	ZnTi		
He et al.^(^ ^26^ ^)^	ZnAl	4-hydroxy-3-methoxybenzoic acid	Anion-exchange and/or co-precipitation
		2-hydroxy-4-methoxybenzophenone-5-sulfonic acid	
		4-hydroxy-3-methoxycinnamic acid	
		4,4’-diaminostilbene-2,2’-disulfonic acid	
		p-aminobenzoic acid	
		Urocanic acid	
Sun et al.^(^ ^27^ ^)^	ZnAl	Cinnamic acid	Co-precipitation
		3,4-dimethoxycinnamic acid	
		p-hydroxycinnamic acid	
Li et al.^(^ ^28^ ^)^	ZnTi	p-aminobenzoic acid	Ion-exchange
Sun et al.^(^ ^29^ ^)^	ZnAl	Cinnamic acid	Co-precipitation
		p-methoxycinnamic acid	
Perioli et al.^(^ ^30^ ^)^	MgAl	p-aminobenzoic acid	Anion-exchange
	ZnAl		
Perioli et al.^(^ ^31^ ^)^	MgAl	2-phenyl-1H-benzimidazole-5-sulfonic acid (Eusolex 232)	Anion-exchange and/or co-precipitation reaction
	ZnAl		
Perioli et al.^(^ ^32^ ^)^	ZnAl	5-benzoyl-4-hydroxy-2-methoxy-benzenesulphonate acid	Ion-exchange
Cursino et al.^(^ ^33^ ^)^	ZnAl	Dodecylsulfate	Co-precipitation
		Dodecylbenzenesulfonate	
Mohsin et al.^(^ ^34^ ^)^	ZnAl	Benzophenone 9	Co-precipitation and ion exchange

LDH: layered double hydroxides; UV: ultraviolet; ZnAl: zinc-aluminium; ZnTi: zinc-titanium; MgAl: magnesium-aluminium.

Li et al.,^(^ ^24^ ^)^ intercalated cinnamic acid, used as a UV absorber, in ZnTi-LDH lamellae through the ion-exchange method. Their objective was to enhance the properties of the active agent, and obtain synergistic effects, considering that zinc and titanium cations are already used in formulations for photoprotection. The techniques used in the characterization showed that the formed product obtained better characteristics relative to the precursors, suggesting better thermal stability and a greater blocking capacity against UV radiation.

In another study by the same group, Li et al.,^(^ ^28^ ^)^ prepared a nanocomposite (organic-inorganic) formed by the intercalation of p-aminobenzoic acid (PABA) in the same type of LDH as mentioned above. The results suggest that the material obtained had the potential to be a safe sunscreen, considering the smaller amount of free radicals (OH and O_2_^-^) generated in comparison with other compounds.

Similarly, Sun et al.,^(^ ^29^ ^)^ investigated nanocomposites obtained by the intercalation of cinnamic acid and p-methoxycinnamic acid in Zn_2_Al-LDH. The method used in the reactions was that of co-precipitation. The authors concluded that the nanocomposites were able to absorb UV radiation and could be considered potent solar protectors.

Cinnamic acid, 3,4-dimethoxycinnamic acid, and p-hydroxycinnamic acid were successfully intercalated in Zn_2_Al-LDH by the co-precipitation method and were investigated for oxidative activity using castor oil as the oxidized material. They showed that oxidation was substantially inhibited and concluded that the LDHs contained the oxidation reaction. Therefore, they hypothesized that these materials acted as radical scavengers.^(^ ^27^ ^)^

In the study of He et al.,^(^ ^26^ ^)^ six molecules, namely, 4-hydroxy-3-methoxybenzoic acid, 2-hydroxy-4-methoxybenzophenone-5-sulfonic acid, 4-hydroxy-3-methoxycinnamic acid, 4,4’- diaminestilbene-2,2’-disulphonic acid, PABA and urocanic acid, were intercalated between Zn_2_Al-LDH lamellae. They showed that all the nanocomposites demonstrated excellent protection against UV rays. In particular, the following three nanocomposites were superior against UVA: Zn_2_Al-LDH/2-hydroxy-4-methoxybenzophenone-5-sulfonic, Zn_2_Al-LDH/4-hydroxy-3-methoxycinnamic acid and Zn_2_Al-LDH/4,4’-diaminostilbene-2,2’-disulphonic acid.

Perioli et al.,^(^ ^30^ ^)^ demonstrated that the intercalation of PABA in LDH represents a new strategy to improve sun protection. The choice of this filter was based on the fact that it is very photo-stable and photosensitive. The results suggested that the filter molecule showed an increase in light stabilization and, by avoiding the direct contact of this molecule with the skin, led to a reduction in skin reactions and allergic problems. Moreover, as the sunscreen interacted well when intercalated in the clay, very low or insignificant release into the skin was demonstrated.

In another study, Perioli et al.,^(^ ^31^ ^)^ used 2-phenyl-1H-benzimidazole-5-sulfonic acid (Eusolex 232) as a sunscreen molecule in LDHs. The storage of the filter in LDHs led to the reduction of the release of the sunscreen, as in the previous study, with consequent increase of its photostability, and avoidance of direct contact with the skin. These results allow us to state that these anionic clays are good matrices for sunscreen formulations. A similar result was obtained by Perioli et al.,^(^ ^32^ ^)^ who intercalated 5-benzoyl-4-hydroxy-2-methoxy-benzenesulfonic acid (4BHF) in LDH.

Cursino et al.,^(^ ^33^ ^)^ showed it was possible to synthesize Zn_x_Al-LDH compounds intercalated with anionic surfactants (dodecylsulfate or dodecylbenzenesulfonate) to promote the adsolubilization of benzophenone (insertion of a neutral species in LDHs). The products showed good adsorption from UVC to UVA, and dermal toxicity tests in rabbits revealed no signs of skin irritation, toxicity or weight loss. In a more recent study, Cursino et al.,^(^ ^35^ ^)^ also performed the adsolubilization of salicylates, cinnamates and benzophenone.

Wang et al.,^(^ ^25^ ^)^ synthesized LDHs containing zinc cations and various amounts of titanium, then evaluated these products for absorption properties. Their results suggested that a higher content of Ti^4+^ was favorable for higher UV radiation absorption capacity, thus indicating that elemental titanium facilitated the absorption of photons and the resulting electron transition. Also, compared with TiO_2_ and ZnO, the ZnTi-LDH product generated fewer active radicals.

Khan et al.,^(^ ^1^ ^)^ confirmed the intercalation of three organic filters, 3,4-dihydroxycinnamic acid (CA), 4-hydroxy-3,5-dimethoxycinnamic acid (SA), and 3-amino-5-trifluoromethylbenzoic acid (FBA) in Zn_2_Al-LDH, using the techniques of X-ray diffraction (XRD) and Fourier-transform infrared spectroscopy (FTIR). They observed that the UV shielding capacity was weak when analyzing the UV-Vis transmittance spectra of LDH. However, all nanohybrids exhibited the ability to absorb UV rays in the UVA range, especially CA/LDH and SA/LDH. Also in this study, they investigated the catalytic oxidation activity of these nanohybrids. The results suggested that LDH contained the catalytic oxidation activity of the intercalated filters and reduced the air oxidation to castor oil.

The thermal stability and UV absorption ability of nanohybrids 2,4-dihydroxybenzophenone-5-sulfonate (DHBS)- ZnA1-LDH were investigated by Li et al.^(^ ^2^ ^)^ The micrograph obtained by scanning electron microscopy shows agglomerates of compact and non-porous structure, which are typical intercalated LDHs, together with the XRD and FTIR techniques that confirmed the success in the synthesis obtained by co-precipitation. Regarding thermal stability, the thermogravimetric data indicate that the starting decomposition temperature of DHBS shifts from 190 to 380°C, increasing the thermal stability of DHBS, in addition to the higher observed UV absorption ability, from 200 to about 500nm.

Mohsin et al.,^(^ ^34^ ^)^ prepared a nanocomposite of benzophenone 9, effective in the protection of UVA rays, and ZnAl-LDH through the co-precipitation and ion exchange method. The objective of the authors was to improve the photostability of the UV filter. X-ray diffraction confirmed the intercalation of the material in both methods, with an increase in basal spacing of 8.8Å to 15.9Å and 16.6Å, respectively. Furthermore, the anions of benzophenone 9 have been released over a long period of time, an important consideration in view of the possibility of controlled release. The cytotoxicity assays performed at the concentrations of 1.562, 3.125, 6.25, 12.5, 25 and 50μg/mL for 24 hours, demonstrated that the products exposed above 25μg/mL caused a reduction of more than 40% in cell viability.

## CONCLUSION

The potential application of layered double hydroxides for photoprotection has led to several studies aimed to investigate the association of organic sunscreens with these inorganic nanocarriers.

Through the reported studies, it is evident that these anionic clays are good matrices for formulations intended for application on the face and body skin for photoprotective purposes. The formation of nanocomposites (organic-inorganic) sunscreen/layered double hydroxides may circumvent the problems related to the use of these sunscreens.
